# Identification and Structure Elucidation of Epoxyjanthitrems from *Lolium perenne* Infected with the Endophytic Fungus *Epichloë festucae* var. *lolii* and Determination of the Tremorgenic and Anti-Insect Activity of Epoxyjanthitrem I

**DOI:** 10.3390/toxins12080526

**Published:** 2020-08-17

**Authors:** Sarah C. Finch, Michèle R. Prinsep, Alison J. Popay, Alistair L. Wilkins, Nicola G. Webb, Sweta Bhattarai, Joanne G. Jensen, Allan D. Hawkes, Jacob V. Babu, Brian A. Tapper, Geoffrey A. Lane

**Affiliations:** 1 Ruakura Research Centre, AgResearch Ltd., Private Bag 3123, Hamilton 3240, New Zealand; alison.popay@agresearch.co.nz (A.J.P.); nikki.webb@agresearch.co.nz (N.G.W.); sweta.bhattarai@agresearch.co.nz (S.B.); joanne.jensen@agresearch.co.nz (J.G.J.); allan.hawkes@agresearch.co.nz (A.D.H.); Jacob@milktest.co.nz (J.V.B.); 2 Chemistry, School of Science, University of Waikato, Private Bag 3105, Hamilton 3240, New Zealand; michele.prinsep@waikato.ac.nz (M.R.P.); a.wilkins@waikato.ac.nz (A.L.W.); 3 Grasslands Research Centre, AgResearch Ltd., Private Bag 11008, Palmerston North 4442, New Zealand; brian.tapper@agresearch.co.nz (B.A.T.); geoff.lane@agresearch.co.nz (G.A.L.)

**Keywords:** epoxyjanthitrem, AR37, endophyte, tremorgen, porina, *Epichloë festucae* var. *lolii*, ryegrass staggers

## Abstract

Epoxyjanthitrems I–IV (**1**–**4**) and epoxyjanthitriol (**5**) were isolated from seed of perennial ryegrass (*Lolium perenne*) infected with the endophytic fungus *Epichloë festucae* var. *lolii.* Although structures for epoxyjanthitrems I–IV have previously been proposed in the literature, this is the first report of a full structural elucidation yielding NMR (Nuclear magnetic resonance) assignments for all five epoxyjanthitrem compounds, and additionally, it is the first isolation of epoxyjanthitriol (**5**). Epoxyjanthitrem I induced tremors in mice and gave a dose dependent reduction in weight gain and feeding for porina (*Wiseana cervinata*), a common pasture pest in New Zealand. These data suggest that epoxyjanthitrems are involved in the observed effects of the AR37 endophyte on livestock and insect pests.

## 1. Introduction

Many of the world’s grasses, including the agriculturally important perennial ryegrass (*Lolium perenne* L.) and tall fescue (*Festuca arundinacea* Schreb.) are infected with *Epichloë* (formerly *Neotyphodium*, formerly *Acremonium*) [1] endophytic fungi. In New Zealand, perennial ryegrass is the most widely sown pasture grass species and under these environmental conditions, the presence of an endophyte is essential for plant productivity and persistence through the expression of beneficial secondary metabolites which provide protection against key pasture pests. Previous research has shown that this beneficial insect interaction is largely driven by the effect of the secondary metabolites, peramine on Argentine stem weevil (*Listronotus bonariensis*) [2] and ergovaline on African black beetle (*Heteronychus arator*) [3]. Unfortunately, in addition to these beneficial secondary metabolites, the naturally-occurring (common-toxic) endophyte-perennial ryegrass association also expresses lolitrem B (**10**), a secondary metabolite which is the cause of ryegrass staggers, a neurological condition of grazing animals responsible for major production losses and farm management issues for farmers [4]. This association also expresses ergovaline, which in addition to its anti-insect effects is responsible for heat stress in grazing animals [5]. 

In an attempt to harness the beneficial effects of endophytes, while minimizing the detrimental ones, a large screening programme has been undertaken whereby endophytes from around the world have been analysed to identify those with a favourable chemical profile. Selection and transfer of these endophytes into grass cultivars has resulted in novel endophyte-grass associations [6]. The first such endophyte to be commercially released in perennial ryegrass was AR1 in 2001 [7]. This combination does not express lolitrem B so is not associated with ryegrass staggers under normal farming practices, but it does produce peramine, so is resistant to Argentine stem weevil. However, AR1 endophyte-infected ryegrass suffers damage by African black beetle and a root aphid (*Aploneura lentisci*), which reduces productivity and persistence of ryegrass [8,9]. Another commercial endophyte, AR37, shows much broader effects against common insect pasture pests. Although not producing peramine and thus being ineffective against adult Argentine stem weevil, AR37 has a strong effect on reducing Argentine stem weevil larval damage [10], has an effect on adult African black beetle [11] as well as effects on porina (*Wiseana cervinata*) [12,13], root aphid [11,14,15] and pasture mealybug (*Balanococcus poae*) [16]. The AR37 endophyte in perennial ryegrass does not express lolitrem B but does induce ryegrass staggers, although episodes recorded in animals grazing AR37 are less frequent and less severe than those observed in animals grazing naturally-occurring, common-toxic endophyte pastures [17]. It has been estimated that the AR37 endophyte will contribute NZ$3.6 billion to the New Zealand economy over the 20-year lifetime of its patent [18]. 

The broad-spectrum insect effects of AR37 are unique among commercial ryegrass endophytes due to their activity against porina and a root aphid, *Aploneura lentisci*, which other endophytes have little or no effect on [8,13]. Porina, the test insect in the bioassay reported here, is the common name given to several species of endemic moths with an annual life cycle that are widely distributed in the South Island and the southern half of the North Island of New Zealand. The larvae live in burrows and feed on a range of pasture plants causing severe damage when populations are high. The effects of AR37 on porina and the root aphid, along with strong activity against Argentine stem weevil and black beetle provides AR37-infected ryegrass with a definite agronomic advantage [15], but to enable these advantages to be captured in future endophytic products, it is important to determine what secondary metabolites are conferring these beneficial effects. Furthermore, the observation of ryegrass staggers in animals grazing this grass-endophyte association, despite the lack of lolitrem B, requires further investigation. According to published information, the expression of epoxyjanthitrems is unique to the AR37 and NEA12 [19] endophytes in perennial ryegrass. Although no full NMR (Nuclear magnetic resonance) structural assignments have been published for these compounds, structures have been proposed in the literature for four out of the five known epoxyjanthitrems [20,21,22]. In AR37 endophyte-infected perennial ryegrass, epoxyjanthitrem I (**1**) is the major compound (typically around 35% of the total epoxyjanthitrems), followed by epoxyjanthitrem III (**3**, 26%), epoxyjanthitrem II (**2**, 11%), epoxyjanthitriol (**5**, 9%) and epoxyjanthitrem IV (**4**, 9%), although it has been observed that these ratios can show a seasonal variation [23]. Because of the structural similarity of these compounds to both lolitrem B and the tremorgenic janthitrems produced by *Penicillium janthinellum* in culture (janthitrems A–D (**6**–**9**), it is hypothesized that epoxyjanthitrems are responsible for the ryegrass staggers induced by AR37 endophyte-infected perennial ryegrass. Due to the instability of janthitrems [24,25], the proof required to confirm this hypothesis has not been previously available. However, a recent study using a mouse bioassay comparing the tremorgenicity of culture-derived janthitrem B (**7**) and janthitrem A (**6**) showed that the 11,12-epoxy group, such as that found in AR37-expressed epoxyjanthitrems, is important for tremorgenic activity [24]. This study also indicated that the epoxy group enhanced the bioactivity of janthitrems against porina larvae. This is consistent with another study that found the concentration of epoxyjanthitrems in AR37 endophyte-infected plant material correlated with activity against porina larvae [26]. These results therefore suggest that the epoxyjanthitrems are important endophyte-expressed metabolites, which contribute to the effects of the AR37 endophyte on both insect pests and animals.

**Figure 1:**
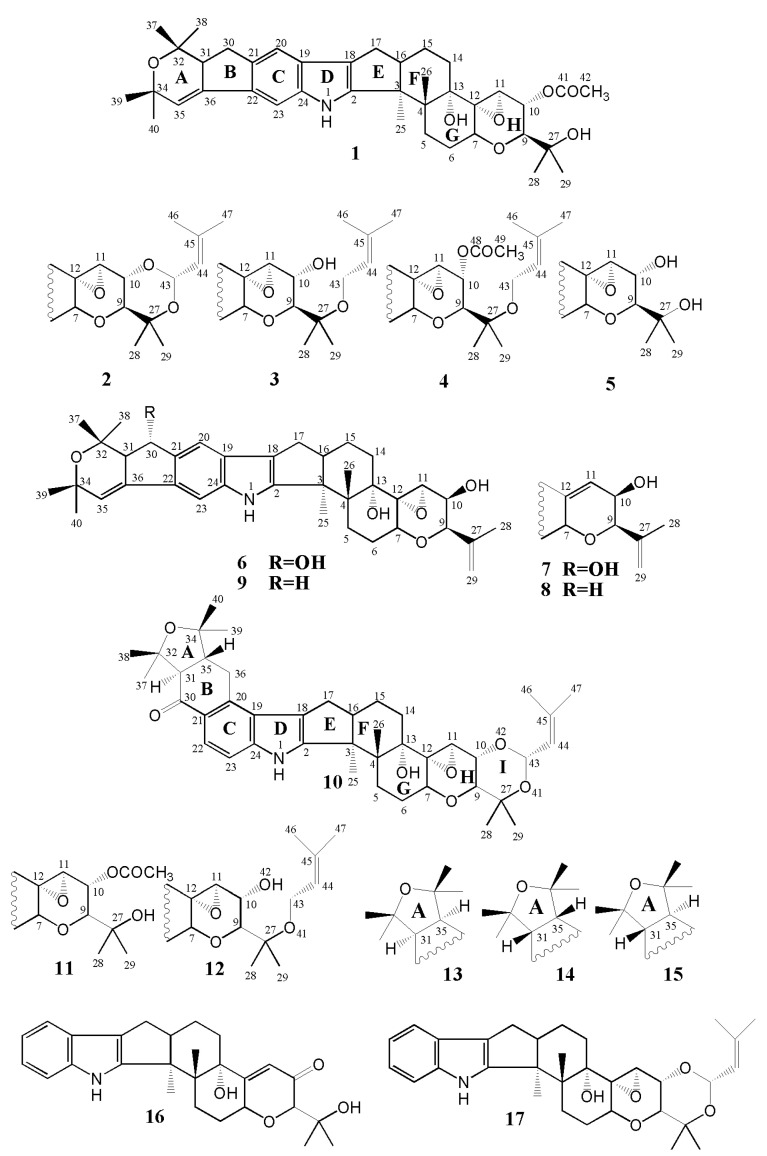
Structures of epoxyjanthitrems I–IV (**1**–**4**), epoxyjanthitriol (**5**), janthitrem A (**6**), janthitrem B (**7**), janthitrem C (**8**), janthitrem D (**9**), lolitrem B (**10**), lolitriol acetate (**11**), lolitrem E (**12**), lolitrem F (**13**), 31-*epi*-lolitrem B (**14**), 31-*epi*-lolitrem F (**15**), paxilline (**16**) and terpendole C (**17**). Note that the numbering system used in this study is one modified by Miles et al. [27] and Babu et al. [24] rather than that used previously for janthitrem compounds [28] to allow direct comparison of NMR chemical shifts of related indole-diterpenoids.

In this study, the instability issues associated with the isolation of pure epoxyjanthitrems from plant material have been overcome, allowing the previously proposed structures for epoxyjanthitrems I–IV to be confirmed, the chemical structure of epoxyjanthitriol to be elucidated, and full NMR assignments to be completed for all of the known epoxyjanthitrems expressed by the AR37-perennial ryegrass association (Figure 1). Testing of the major compound, epoxyjanthitrem I, on porina larvae and in the mouse bioassay for tremorgenicity, has shown that this class of compound is active against both insects and animals.

## 2. Results and Discussion

### 2.1. Structure Elucidatation

The structure of epoxyjanthitrem I was determined by analysis of its ^1^H (Figures S1–S3), ^13^C NMR (Figures S4–S6) and mass spectra along with the results of COSY, NOESY, ^1^H-^13^C HSQC and ^1^H-^13^C HMBC NMR experiments. HRESIMS of epoxyjanthitrem I (positive ion mode) contained a peak at m/z 668.3536 [M + Na]^+^, consistent with a molecular formula of C_39_H_51_NO_7_ (Figure S7). 

The ^1^H NMR spectrum of epoxyjanthitrem I in acetone-d6 (Table 1, Figures S1–S3) included a singlet at 9.86 ppm attributable to an N-H proton, two aromatic singlets at 7.39 and 7.18 ppm, an olefinic proton signal at 5.93 ppm and two aliphatic methine proton signals at 2.83 and 2.81 ppm, the chemical shifts of which suggested that they were bridgehead protons. The spectrum also included resonances attributable to four methine protons attached to oxygenated carbons (5.17, 4.20, 3.55 and 3.48 ppm), eight aliphatic methyl group resonances and a number of aliphatic methylene proton signals. The presence of an acetyl methyl proton signal at 2.05 ppm (solvent obscured in the ^1^H NMR spectrum) was revealed in the HSQC spectrum of epoxyjanthitrem I.

The ^13^C NMR spectrum of epoxyjanthitrem I in acetone-d6 (Table 2, Figures S4–S6) contained 39 signals, including 24 protonated carbon resonances. These protonated resonances comprised two aromatic signals (114.3 and 104.0 ppm), an olefinic carbon resonance at 119.6 ppm, four oxygenated methine carbon resonances (76.6, 72.3, 68.7 and 62.3 ppm), two bridgehead methine carbon signals (50.9 and 49.8 ppm), six methylene carbon resonances (33.5, 30.3, 28.7, 27.8, 26.9 and 21.5 ppm), one acetyl methyl carbon signal at 21.2 ppm and eight aliphatic methyl carbon signals (32.4, 30.6 (2), 26.7, 26.6, 22.4, 18.8, 16.5). The spectrum also contained 15 quaternary carbon signals, comprising an acetyl carbonyl resonance at 170.5 ppm, six aromatic signals (154.9, 141.3, 140.9, 133.6, 127.7 and 116.8 ppm), an olefinic carbon resonance at 136.9 ppm, five oxygenated carbon signals (78.0, 74.7, 72.9, 71.4 and 70.4 ppm) and two bridgehead carbon resonances at 51.7 and 43.2 ppm). Atom connectivities were established by COSY, NOESY, ^1^H-^13^C HSQC and ^1^H-^13^C HMBC NMR experiments. The results of these experiments allowed the structure of epoxyjanthitrem I to be assembled, confirming the presence of a skeleton that represented a combination of the structural features of janthitrem C (**8**) and lolitriol acetate (**11**). Epoxyjanthitrem I has the same skeleton as janthitrem C in rings A-G but contains a modified ring H. Ring H was found to be identical to that of the analogous ring in lolitriol acetate. The C11-C12 double bond of janthitrem C is replaced by an epoxide group in epoxyjanthitrem I and the isopropene moiety at C9 and the hydroxyl group at C10 are replaced by an hydroxyisopropyl substituent and an O-acetyl group, respectively. HMBC correlations from H9 to C27, H28 and H29 to C9, and H10 to C41 confirmed the positions of these substituents in ring H. NOE correlations supported the relative configuration as shown in epoxyjanthitrem I. For example, NOE correlations between H7 and H9, between H10 and H11 and between H16 and H26 indicated that epoxyjanthitrem I possessed the same relative configuration as janthitrem C [25] in rings E-G and as lolitriol acetate [29] in ring H. Additionally, the J_H9-H10_ coupling constant (9.4 Hz) of epoxyjanthitrem I was in agreement with that reported for lolitriol acetate (9.4 Hz) [29], confirming the foregoing configurational assignment. The ^1^H and ^13^C NMR spectral data of epoxyjanthitrem I in acetone-d6 (Table 1 and Table 2) were in good agreement with literature data for the relevant structural parts of janthitrem C [25] and lolitriol acetate [29]. 

The ^1^H and ^13^C NMR spectra of epoxyjanthitrems II–IV and epoxyjanthitriol in acetone-d6 (Table 1 and Table 2) indicated that these compounds shared an identical structure to that of epoxyjanthitrem I in rings A–G and all structural differences concerned the substituents in ring H of the compounds. HRESIMS of epoxyjanthitrem II in positive ion mode contained a peak at m/z 692.3927 [M + Na]^+^ consistent with a molecular formula of C_42_H_55_NO_6_. The ^1^H and ^13^C NMR spectra of epoxyjanthitrem II in acetone-d6 (Table 1 and Table 2) displayed some key differences to those of epoxyjanthitrem I. The spectra lacked resonances for the C10 O-acetyl group (carbonyl and acetyl methyl carbon and acetyl methyl proton signals) but contained additional signals. The ^1^H NMR spectrum of epoxyjanthitrem II contained two mutually coupled methine proton doublets at 5.58 and 5.17 ppm (*J* = 6.2 Hz) and two additional methyl resonances at 1.69 and 1.70 ppm, whilst the ^13^C NMR spectrum contained additional methine carbon signals at 124.0 and 93.3 ppm and two additional methyl carbon signals at 18.8 and 25.5 ppm. An HSQC correlation connected the methine proton resonance at 5.58 ppm to the carbon signal at 93.3 ppm, chemical shifts which are characteristic of a dioxygenated carbon and its attached proton. HSQC correlations connected the methine proton at 5.17 ppm (H44) to the carbon at 124.0 ppm (C44) and the methyl proton resonances at 1.69 and 1.70 ppm (H46 and H47) to the methyl carbon signals at 18.8 and 25.5 ppm, respectively (C46 and C47). HMBC correlations from H43 to the olefinic carbon at 138.1 ppm (C45), H44 to C46 and C47 and H46 to C44, C45 and C47 confirmed the presence of an isoprene unit in epoxyjanthitrem II. Key HMBC correlations from H10 to C43 and H43 to C27, confirmed that this unit was attached to the oxygen atoms on C10 and C27, forming a six-membered ring (ring I) as found in lolitrem B. All of these data together indicated that epoxyjanthitrem II had an identical structure to epoxyjanthitrem I and janthitrem C in rings A–G and that rings H–I were identical to rings H–I in lolitrem B [30]. NOE correlations between H10 and H43 and between H29 and both H10 and H43 confirmed that the stereochemistry around ring I in epoxyjanthitrem II was the same as that of lolitrem B [30]. This was also consistent with the close match of the J_H9-H10_ coupling constant (9.3 Hz) of epoxyjanthitrem I and that reported for lolitrem B (9.5 Hz) [30]. The ^1^H and ^13^C NMR spectral data of epoxyjanthitrem II in acetone-d6 (Table 1 and Table 2) were in good agreement with the literature data for the relevant structural parts of janthitrem C [25] and lolitrem B [30]. 

HRESIMS of epoxyjanthitrem III (**3**) in positive ion mode contained a peak at m/z 694.4063 [M + Na]^+^ consistent with a molecular formula of C_42_H_57_NO_6_, indicating that the structure of epoxyjanthitrem III contains two additional hydrogens to that of epoxyjanthitrem II. The ^1^H and ^13^C NMR spectra of epoxyjanthitrem III in acetone-d6 (Table 1 and Table 2) were very similar to those of epoxyjanthitrem II but with some key differences. The methine proton doublet signal at 5.58 ppm in the ^1^H NMR spectrum of epoxyjanthitrem II (H43) was replaced by a doublet of doublets at 4.03 ppm that integrated as 2 protons. The methine carbon resonance at 93.3 ppm (C43) was similarly absent and replaced by a methylene carbon signal at 58.8 ppm. Additionally, the multiplicity of H44 had changed from a doublet to a triplet, the signal had moved downfield from 5.17 pm in epoxyjanthitrem II to 5.26 ppm in epoxyjanthitrem III and other chemical shift differences were present in nearby proton and carbon signals. For example, H10 shifted upfield from 4.04 to 3.95 ppm and C10 from 71.8 ppm to 68.0 ppm. While the H10 signal had correlations in the HMBC spectrum to C9, C12 and C27 as found in epoxyjanthitrem II, the correlation to C43 was absent. All of these data indicated that epoxyjanthitrem III lacked the ring I of epoxyjanthitrem II but shared an identical structure around ring H to lolitrem E (**12**) [31], which was confirmed by selective NOE (SELNOESY) experiments. For example, irradiation of H9 predominantly enhanced H7 and the H13 hydroxyl proton and irradiation of H11 enhanced H10. The J_H9-H10_ coupling constant (9.1 Hz) was comparable to those of lolitrem B (9.5 Hz) [30] and lolitrem E (9.6 Hz) [31], further confirming the C9 stereochemistry. The ^1^H and ^13^C NMR spectral data of epoxyjanthitrem III in acetone-d6 (Table 1 and Table 2) were in good agreement with literature data for the relevant structural parts of janthitrem C [25] and lolitrem E [31]. 

HRESIMS of epoxyjanthitrem IV (**4**) in positive ion mode contained a peak at m/z 736.4201 [M + Na]^+^ consistent with a molecular formula of C_44_H_59_NO_7_, indicating that the structure of epoxyjanthitrem IV contains two additional carbon and hydrogen atoms and one additional oxygen atom over that of epoxyjanthitrem III. The ^1^H and ^13^C NMR spectra of epoxyjanthitrem IV in acetone-d6 (Table 1 and Table 2) reflected these changes but were otherwise very similar to those of epoxyjanthitrem III. Additional carbon signals at 179.2 and 21.2 ppm and an additional methyl proton singlet at 1.99 ppm were attributed to the presence of an O-acetyl group as in epoxyjanthitrem I and an HMBC correlation from H10 to the carbonyl carbon confirmed its placement at C10 as in epoxyjanthitrem I. Although the spectra of epoxyjanthitrem IV and epoxyjanthitrem III were very similar, there were some differences in the chemical shifts of protons and carbons near the acetate in epoxyjanthitrem IV as expected. For example, the most obvious differences in the spectra of epoxyjanthitrem IV from those of epoxyjanthitrem III were a large downfield shift of H10 from 3.95 ppm in epoxyjanthitrem III to 5.26 ppm in epoxyjanthitrem IV, attributable to proximity to the O-acetyl group as in epoxyjanthitrem I and an upfield shift of C27 from 79.5 ppm in epoxyjanthitrem III to 76.8 ppm in epoxyjanthitrem IV. Other notable differences between the spectra of epoxyjanthitrem IV and epoxyjanthitrem III were in the chemical shifts of C28 and C29 and H28 and H29. In epoxyjanthitrem III, these shifts occurred at 23.9 (C28) and 1.28 ppm (H28) whereas they occurred at 24.5 (C28) and 1.20 ppm (H28) in epoxyjanthitrem IV, at 19.7 (C29) and 1.25 ppm (H29) in epoxyjanthitrem III and at 21.4 (C29) and 1.18 ppm (H29) in epoxyjanthitrem IV. Thus, the substituents on ring H of epoxyjanthitrem IV were an O-acetyl group on C10 as in epoxyjanthitrem I and an isoprene group at C27 as in epoxyjanthitrem III. NOE correlations (for example from H9 to N7, H10 and H28 and H10 to H9 and H11) and the magnitude of the J_H9-H10_ coupling constant (9.3 Hz) confirmed that the stereochemistry was the same as that in epoxyjanthitrem III. The ^1^H and ^13^C NMR spectral data of epoxyjanthitrem IV in acetone-d6 (Table 1 and Table 2) were in good agreement with the literature data for the relevant structural parts of epoxyjanthitrem I, epoxyjanthitrem III and lolitrem E [31]. 

HRESIMS of epoxyjanthitriol in positive ion mode contained a peak at m/z 604.3633 [M + H]^+^ consistent with a molecular formula of C_37_H_49_NO_6_, indicating that the structure of epoxyjanthitriol contains two less carbon and hydrogen atoms and one less oxygen atom compared to that of epoxyjanthitrem I. The ^1^H and ^13^C NMR spectra of epoxyjanthitriol in acetone-d6 (Table 1 and Table 2) reflected these changes but were otherwise very similar to those of epoxyjanthitrem I. For example, the carbonyl and methyl carbon signals of epoxyjanthitrem I and the corresponding acetate methyl proton signal were absent in epoxyjanthitriol and although a signal was not observed for the C10 hydroxyl proton, the chemical shifts of C10 and H10 of epoxyjanthitriol (68.3 and 3.99 ppm, respectively) were very similar to those of C10 and H10 of epoxyjanthitrem III (68.0 and 3.95 ppm, respectively), indicating that a hydroxyl group was present at C10 in epoxyjanthitriol rather than an O-acetyl group as in epoxyjanthitrem I. Other chemical shift differences between epoxyjanthitrem I and epoxyjanthitriol, which are reflective of these structural differences, were the chemical shifts of C27-C29 and of H28-H29 in epoxyjanthitriol compared to those of epoxyjanthitrem I. The magnitude of the J_H9-H10_ coupling constant in epoxyjanthitriol (9.0 Hz) was close to that in epoxyjanthitrem I (9.4 Hz), and this, along with NOE correlations from H9 to H7 and H10 and from H10 to H11, confirmed that the stereochemistry was the same as that in epoxyjanthitrem I. Other than the expected differences from the lack of an acetyl group at C10, the ^1^H and ^13^C NMR spectral data of epoxyjanthitriol in acetone-d6 (Table 1 and Table 2) were in good agreement with those of epoxyjanthitrem I. 

The structural differences between the epoxyjanthitrem compounds all pertained to the right hand side of the molecule involving either variation of the substituent at C10 and/or the presence/absence of an isoprene unit attached to the C27 hyroxyl group. Full NMR data for the epoxyjanthitrems are presented in Table 1 and Table 2. 

### 2.2. Insect Bioassay

Significant effects of epoxyjanthitrem I were found on the amount of diet consumed over each 24 h period and the weight change of the porina larvae over the 7 days of the experiment. These effects were reflected in differences in scores for frass production and the amount of webbing produced by larvae, but these data are not presented.

Analysis of porina diets at time zero showed that, for the experiment incorporating pure epoxyjanthitrem I into insect diets, the concentrations were as expected (average of 98%) showing that epoxyjanthitrem I was stable during the diet-making process. However, analysis of diets after 24 h showed some decomposition of epoxyjanthitrem I over this time period (average of 81% of the starting concentration).

**Figure 2:**
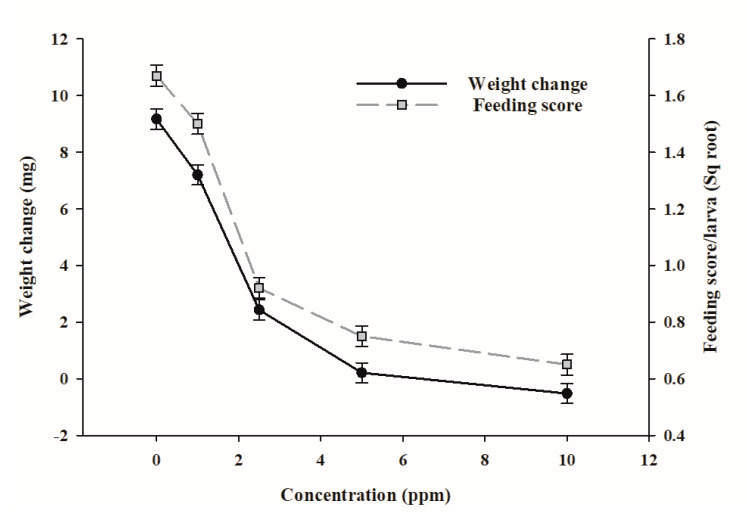
Weight change and mean daily feeding (scored on a scale of 0–10 and then square root transformed for analysis) for porina larvae (n = 20) fed diets containing five different concentrations of epoxyjanthitrem I over 7 days. Error bars are ± SE.

Average weight of larvae at the beginning of the experiment was 14.8 mg with a range of ±0.5 mg across treatments. Survival of larvae during the experiment was 95% in the solvent control and 100% in treatments containing epoxyjanthitrem I. The average feeding score for porina fed epoxyjanthitrem I at 1 ppm for 7 days did not differ significantly from the solvent control but was significantly (*p* < 0.001) inhibited at concentrations of 2.5, 5 and 10 ppm (Figure 2). Weight change also declined with increasing concentration at the same rate as the feeding scores (Figure 2) and was significantly reduced (*p* < 0.001) relative to the solvent control at all concentrations except 1 ppm. However, weight gain as a percentage of the initial weight was significantly lower for larvae fed epoxyjanthitrem I at 1 ppm compared with those fed the solvent control (1 ppm epoxyjanthitrem I 51.1%; solvent control 63.4%, *p* < 0.05), suggesting some activity even at this low concentration. Webbing, produced by insects as a means of protection and a good indicator of porina health, followed a very similar pattern of scores (data not presented) indicating that this was strongly linked to the deterrent effect of the diet and reduced consumption. 

The diet ingredients are mainly carrot and clover for which the moisture content varies resulting in a slightly variable dry weight of the diet. However, the dry weight of the diet is generally between 9 and 11% of the wet weight. At a median of 10%, the 2.5 ppm concentration of epoxyjanthitrem I is equal to a 25 ppm dry weight concentration which had a highly significant effect on feeding and weight change of porina in this experiment. There was also some effect detectable at a wet weight concentration of 1 ppm (equivalent to 10 ppm dry weight concentration) indicating that the threshold for activity of epoxyjanthitrem I may lie between 10 and 25 ppm dry weight. Although our study tested only epoxyjanthitrem I, the activity of the other epoxyjanthitrem compounds are also likely to contribute to the overall effects of AR37 on this insect. Using freeze-dried AR37-infected ryegrass incorporated into an agar diet [26] showed that antifeedant effects could occur at a dry weight concentration of total epoxyjanthitrems as low as 14 ppm. This is well within the concentrations that are typically observed in the field although concentrations of endophyte-expressed alkaloids is weather dependant. It has been reported that in New Zealand under moist, cool conditions in 2012, the total epoxyjanthitrem concentrations ranged between 18.5 and 26.0 ppm in the January to March period, and in 2013 when it was warmer and drier, they ranged from 27.0 to 50.5 in February and March [32].

### 2.3. Mouse Bioassay

**Figure 3:**
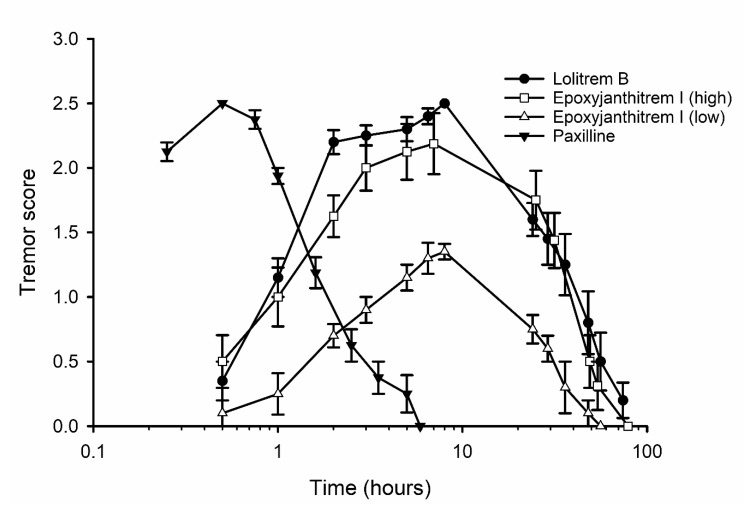
Tremor score versus time for groups of mice (n = 4) dosed intraperitoneally with paxilline (6 mg/kg), lolitrem B (2 mg/kg), epoxyjanthitrem I (high, 14 mg/kg) or epoxyjanthitrem I (low, 8 mg/kg). Tremor scores for the control group were zero at all time points. Error bars represent the standard error of the means.

Initially, epoxyjanthitrem I was dosed at 8 mg/kg, but this was found to give only a low tremor response. Subsequently, groups of four mice were dosed with epoxyjanthitrem I at 14 mg/kg, lolitrem B at 2 mg/kg, paxilline (**16**) at 6 mg/kg or with solvent alone. A tremor score of zero was recorded for control mice at every time point, but a tremor response was observed in all of the other treatment groups (Figure 3). The time course of action for epoxyjanthitrem I was found to be unlike that of paxilline or for those reported for janthitrems and the epoxyjanthitrem isolated from *P. janthinellum* [24]. In these cases, tremors peaked at 30 mins post-dosing, and the mouse had completely recovered by 6 h post-dosing. In contrast, mice dosed with either epoxyjanthitrem I or lolitrem B showed tremors peaking at 7–8 h post-dosing which persisted for over 2 days. 

Recent work comparing the impact of adding an 11,12-epoxy group (janthitrem A, (**6**)) to that of janthitrem B (**7**) showed it to increase the severity of tremor, but the time course of action for both compounds was of short duration [24]. Due to the similarity in the structures of epoxyjanthitrem I and janthitrem A, both containing 11,12 epoxy-groups, it was surprising to observe a long duration tremor for epoxyjanthitrem I. This is the first time any indole-diterpenoid other than those of the lolitrem class has shown this sustained tremor effect in the mouse bioassay.

Comparing the structures of epoxyjanthitrem I and janthitrem A, only the latter compound contains a 30-OH group. However, early reports list janthitrem C (**8**), which also lacks the 30-OH group, to be similarly tremorgenic to janthitrem B [33], making it unlikely that this structural feature is the cause of the altered tremor response. It is more likely that the stereochemistry of the functional groups at C10 is the key difference between those janthitrems isolated from the AR37-perennial ryegrass association, which are all α-orientated at this position, and those isolated from *P. janthinellum*, which are all β-orientated. Further strengthening this case is the known importance of the 13α-OH group for tremorgenicity as no known indole-diterpene lacking a 13α-OH group has been found to be tremorgenic. Furthermore, a study comparing the structural isomers lolitrem B, lolitrem F (**13**), 31-epi-lolitrem B (**14**) and 31-epi-lolitrem F (**15**), which differ only in the stereochemistry of H31 and H35 at the A/B ring junction (Figure 1), showed that 31-epi-lolitrem B was the only compound to be non-tremorgenic. Molecular modelling of these four compounds showed that the non-tremorgenic compound, (31-epi-lolitrem B), was also the only one where the A/B rings protruded onto the α-face of the molecule [34]. This observation, along with the demonstrated importance of the 13α-OH group, led the authors to believe that the α-face of these compounds is important for the induction of tremorgenicity. This is consistent with our finding that the stereochemistry of the C10 substituents is important, with only those that are α-oriented generating the long-duration tremor in the mouse bioassay. However, the structure-activity relationships of these indole-diterpenoid compounds is further complicated by the observation that terpendole C (**17**) [35,36] induces tremors of only short duration [37]. The only difference between this compound and that of the potent tremorgen, lolitrem B, is the lack of the A/B rings found in lolitrem B (Figure 1). This demonstrates that additional rings are required at the left-hand end of the indole-diterpenoid structure to induce long-duration tremors. In addition to the A/B rings of the lolitrem class of compounds, we can now confirm that the A/B rings of the epoxyjanthitrem class of compounds are sufficient to induce this sustained tremor response. Although some important structural features have been identified for tremorgenicity, the structure-activity relationships are too complex to be able to predict tremorgenic activity on the basis of chemical structure alone, and as each novel compound is tested, our understanding will grow.

The fact that epoxyjanthitrem I exhibited the sustained tremor response characteristic of lolitrem B, the known cause of ryegrass staggers in livestock grazing common-toxic, endophyte-infected pastures, makes it highly likely that the epoxyjanthitrems are responsible for the ryegrass staggers seen in livestock grazing AR37 endophyte-infected pastures. However, although epoxyjanthitrem I has been proven to induce tremorgenicity using the mouse model in the current study, the impact of the other epoxyjanthitrems on animals is still unknown. Both epoxyjanthitrem I and lolitrem B induce the long-duration tremor response in mice, but a similar severity of tremor was generated by 2 mg/kg of lolitrem B and 14 mg/kg of epoxyjanthitrem I, which demonstrates that lolitrem B is a much more potent tremorgen. This is consistent with the observation that ryegrass staggers induced by AR37 endophyte-infected pastures tends to be less frequent and of reduced severity compared to that induced by common-toxic, endophyte-infected pastures [17] and that when ryegrass staggers is observed, the concentrations of epoxyjanthitrems are much higher than those of lolitrem B. In a study where sheep were grazing side-by-side in paddocks of common-toxic and AR37 endophyte-infected ryegrass, concentrations of 3.4 ppm lolitrem B induced more severe staggers than 35.7 ppm total (sum of **1**–**5**) of epoxyjanthitrems [20]. Similarly, dairy cows were observed to develop ryegrass staggers when fed herbage containing 1.8 ppm lolitrem B whereas cows fed herbage containing 14.6 ppm total epoxyjanthitrems showed no signs of ryegrass staggers and in fact, ryegrass staggers have never been recorded in dairy cows grazing pasture infected with the AR37 endophyte [38].

## 3. Conclusions

In this study, full NMR assignments were determined for the five epoxyjanthitrems expressed by AR37 endophyte-infected perennial ryegrass. The testing of epoxyjanthitrem I in a mouse bioassay showed it to elicit a tremor response of an analogous time course of action to that of lolitrem B, although it was much less potent. This strongly suggests that the epoxyjanthitrem class of compounds is responsible for the ryegrass staggers seen in livestock grazing this endophyte-grass association. Furthermore, the testing of epoxyjanthitrem I on porina showed it to be bioactive, demonstrating that this class of compound is involved in the unique effects of the AR37 endophyte against insect pests.

## 4. Materials and Methods

### 4.1. Epoxyjanthitrem Analysis by Analytical HPLC

For the analysis of seed for epoxyjanthitrems, ground seed (25 mg) was extracted with acetone (1.5 mL) using an over-over mixer at 30 rotations/min for 1 h. The extract was then centrifuged (5 min, 5600× *g*, Eppendorf, Hamburg, Germany) and analysed by HPLC. Insect diets were analysed for epoxyjanthitrems by extracting insect diets (50 mg) with acetone (1 mL) using an over-over mixer as above for 2 h. The extract was centrifuged as above and analysed by HPLC. To prevent degradation of epoxyjanthitrems, the extractions were performed in the dark. Epoxyjanthitrems were quantified by comparison with a standard of epoxyjanthitrem I shown to be at least 95% pure by nuclear magnetic resonance (NMR) spectroscopy. For analysis of fractions resulting from the isolation of epoxyjanthitrems, as well as extracts of seed and insect diets, a 4.6 × 250 mm ODS column (Phenomenex, Torrance, CA, USA) fitted with a 4 × 3 mm Phenomenex SecurityGuard^TM^ containing two C_18_ cartridges (Torrance, CA, USA) was used with an eluent of water-acetonitrile (1:19, 1 mL/min). Eluting compounds were detected using either an Agilent Series 1100 fluorescence detector or a Shimadzu RF-10A fluorescence detector (excitation at 333 nm, emission detection at 385 nm). 

### 4.2. Isolation of Epoxyjanthitrem I for Testing on Insects and Mice 

Petroleum ether was used as the extraction solvent for the generation of epoxyjanthitrem I for testing on insects and mice since this solvent did not extract epoxyjanthitriol, which led to a quicker and easier isolation of epoxyjanthitrem I. In contrast, since all epoxyjanthitrems were needed for the structural elucidation work, acetone was used as the extraction solvent, resulting in a more comprehensive preparative-HPLC purification approach.

Epoxyjanthitrems were extracted from ground AR37-infected perennial ryegrass seed (cv. Extreme sourced from PGG Wrightson Seeds Limited, Christchurch, New Zealand) (70 g) with petroleum ether (40–60 °C, 450 mL) by Soxhlet extraction for 3 h. After the extraction period, the seed was replaced with fresh seed (70 g), which was extracted for a further 3 h. This process was repeated twice more to yield an extract resulting from 280 g of seed. The petroleum ether was then extracted with acetonitrile (2 × 200 mL), which was dried under reduced pressure to yield an epoxyjanthitrem fraction. Epoxyjanthitrems were found to be highly unstable, so to minimize degradation, the fractions were kept on ice and were protected from light during all steps of the purification process. 2-Mercaptoethanol was also added to eluents at some stages of the purification as an anti-oxidant. To separate epoxyjanthitrem I from epoxyjanthitrems II–IV, flash column chromatography using silica gel (Merck, Art. 9385, Branchburg, NJ, USA) was utilized. The sample was applied to a silica column (4 × 15 cm) in toluene which was then eluted with a gradient of toluene-acetone (100% toluene, 100 mL; 90% toluene, 200 mL; 85% toluene, 200 mL; 80% toluene, 200 mL; 75% toluene, 200 mL; 70% toluene, 200 mL; 50% toluene, 200 mL; 0% toluene, 200 mL). Fractions (13 mL) were collected and analysed for epoxyjanthitrems which showed that epoxyjanthitrems II–IV eluted in the 90% toluene eluent whilst epoxyjanthitrem I eluted in the 85, 80 and 75% toluene eluent.

To further purify the fraction containing epoxyjanthitrem I, two additional silica flash columns were run (2.5 × 12 cm), one with an eluent of 17:3 toluene-acetone with the addition of 0.25% 2-mercaptoethanol and one with an eluent of 23:2 toluene-acetone, again with the addition of 0.25% mercaptoethanol. For each column, fractions were collected and analysed by HPLC. The fractions found to contain epoxyjanthitrem I were then combined and dried down under reduced pressure. To further purify epoxyjanthitrem I, a C_18_ Sep-Pak was used (Waters Corporation, Milford, MA, USA). The Sep-Pak was firstly flushed with acetonitrile (4 mL) and the sample applied in acetonitrile (0.5 mL). Acetonitrile (2 mL) was eluted and collected as the epoxyjanthitrem fraction followed by acetonitrile (1 mL) and acetone (3 mL) into waste. Aliquots (0.5 mL) of the total sample (2.5 mL) were processed in this way and the 2 mL acetonitrile fractions combined to yield the fraction containing epoxyjanthitrem I. This process was repeated following the same protocol to remove further contaminants. The final purification of epoxyjanthitrem I was achieved using two preparative HPLC procedures performed using a Waters series 600 controller and a Prodigy 5 µm ODS (3) HPLC column (250 × 10 mm) (Phenomenex, Torrance, CA, USA). Epoxyjanthitrems were detected at 265 nm using a Waters 486 UV detector. The first procedure was conducted using an eluent of 19:1 acetonitrile-water at 5 mL/min. The peak representing epoxyjanthitrem I was detected by UV spectroscopy and was collected in a flask containing 2-mercaptoethanol. Aliquots of the sample were applied until the entire sample had been processed in this way. The resulting fraction containing epoxyjanthitrem I was dried down before conducting the second preparative HPLC procedure. In this case, the same chromatographic equipment was used, but this time, the eluent utilized was 100% methanol at 4 mL/min, and 2-mercaptoethanol was not added to the collection flask. The resulting epoxyjanthitrem I was analysed by NMR spectroscopy which showed it to be of >95% purity. Full ^1^H and ^13^C NMR assignments are presented in Table 1 and Table 2. An accurate weight was then obtained (2.60 mg) and aliquots prepared for testing on insects and mice.

### 4.3. Insect Bioassay

Six week-old porina larvae reared from eggs [39] were starved overnight and then weighed individually. Larvae were assigned to replicates so that all larvae within a replicate were of a similar weight but randomised amongst treatments. The 20 replicate larvae per treatment were each placed in specimen containers (75 mL) half filled with commercially available fine bark chips. 

An agar-based semi-synthetic diet [39] without the antimicrobials was prepared and epoxyjanthitrem I, dissolved in 100 µL DMSO, was added to 10 g of diet. Four concentrations of epoxyjanthitrem I were prepared (1, 2.5, 5, 10 µg/g wet weight) along with a DMSO control. Discs of diet (7 mm diam.) were taken and placed on top of the bark, and the lids were replaced. The experiment was kept in the dark at 15 °C. After 24 h, each diet was visually scored for feeding on a scale of 0–10 where 0 = no feeding and 10 = all diet consumed. The amount of webbing present was also scored on a scale of 0–5 where a score of 0 represents no webbing present, and a score of 5 represents considerable webbing which encases diet and faecal material. The diet disc was replaced daily with fresh diet for 7 days. Each time diet was replaced, remnant samples of the diet that the porina had fed on were taken as well as a sample of the fresh diet to analyse for epoxyjanthitrem I. Porina were reweighed at the completion of the trial.

The feeding score data were normalised by square root transformation before a repeated measures analysis was carried out using REML with treatment and date as factors. The daily webbing score and weight change of larvae over 7 days were analysed using ANOVA, blocked by replicate, to make pairwise comparisons for each concentration with the solvent control at each date. 

### 4.4. Mouse Bioassay

Pure lolitrem B and paxilline were obtained using the methods detailed Miles et al. [31] and Munday-Finch et al. [40], respectively. Epoxyjanthitrem I, lolitrem B and a related indole-diterpene, paxilline [41] (Figure 1) were administered by intraperitoneal injection as DMSO/water solutions (9:1, 50 µL) into mice (Swiss, female, weight 25 ± 3g). Control mice were injected with the solvent alone. Dose rates which induce an acceptable maximum tremor response are well established for lolitrem B [42] (2 mg/kg) and paxilline [27] (6 mg/kg). A starting dose rate of 8 mg/kg was used for epoxyjanthitrem I, which gave only a low tremor response; a dose rate of 14 mg/kg was therefore subsequently used for the experiment. Groups of four mice were used for each treatment group. All animal manipulations were approved by the AgResearch Ruakura (Hamilton, New Zealand) Animal Ethics Committee established under the Animal Protection (code of ethical conduct) Regulations Act, 1987 (New Zealand), (project number 14454, approval date 03 May 2018). Tremor score was rated 1–5 on the basis of a visual rating scale using well established protocols [42,43]. Tremors were measured regularly until the mice had completely recovered. As indicated by the log scale of the time axis in Figure 3 tremors following the injection of either lolitrem B or epoxyjanthitrem I rose quickly for the first 8 h before slowly receding over the next 3 days. The time intervals between tremor measurements reflect this progression and subsidence of tremors. In contrast, the tremors induced by paxilline rose and receded quickly and consequently the times between tremor measurements were much shorter.

### 4.5. Isolation of Epoxyjanthitrems for Structural Elucidation

Epoxyjanthitrems were extracted from ground AR37-infected perennial ryegrass seed (cv. Base sourced from PGG Wrightson Seeds Limited, Christchurch, New Zealand) (70 g) with acetone (450 mL) by soxhlet extraction for 7 h. The extract was then filtered and dried under reduced pressure. The resulting solid was dissolved in methanol (150 mL) and water (12.5 mL) and sodium chloride (saturated solution, 0.5 mL) added. The methanol-water solution was extracted three times with petroleum ether (40–60 °C, 100 mL) which was discarded and the methanol-water dried under reduced pressure to yield the epoxyjanthitrem fraction. This fraction was dissolved in methanol (10 mL), water added (3.3 mL) and applied to a C_18_ flash column (2.5 × 4 cm) which was eluted with 3:1 methanol-water (35 mL) into waste followed by 9:1 methanol-water (450 mL) to elute the epoxyjanthitrems. The epoxyjanthitrems fraction was dried under reduced pressure before redissolving in 9:1 toluene-acetone (3 mL) which was applied to a silica gel column (2.5 × 20 cm) and eluted with a gradient of toluene-acetone (100% toluene, 100 mL; 85% toluene, 600 mL; 80% toluene, 200 mL; 50% toluene, 100 mL; 0% toluene, 100 mL). Fractions (13 mL) were collected and analysed for epoxyjanthitrem content which showed that a mixture of epoxyjanthitrem I and epoxyjanthitriol eluted in the first 105 mL of the 85% toluene eluent and a mixture of epoxyjanthitrems II–IV eluted in the following 195 mL of the same eluent. These two fractions were dried down under reduced pressure. This entire extraction and initial purification process were conducted on three more batches of seed (70 g each) to yield a bulk fraction containing epoxyjanthitrem I and epoxyjanthitriol and a bulk fraction containing epoxyjanthitrems II–IV, which were each then further purified by preparative HPLC. This was performed using a Shimadzu LC-20AD controller and a Prodigy 5 µm ODS (3) HPLC column (250 × 10 mm) (Phenomenex, Torrance, CA, USA) fitted with a 10 × 10 mm Phenomenex SecurityGuard^TM^ (Torrance, CA, USA). Epoxyjanthitrems were detected with a Shimadzu RF-20A fluorescence detector (excitation at 333 nm, emission detection at 385 nm). Initial removal of contaminants from the mixture of epoxyjanthitrem I and epoxyjanthitriol was achieved using an eluent of 19:1 acetonitrile-water, 10 mL/min. The two epoxyjanthitrem compounds were then separated using an eluent of 9:1 acetonitrile-water, 10 mL/min. Epoxyjanthitrems II–IV were separated using an eluent of 100% acetonitrile, 10 mL/min. Using this solvent system, epoxyjanthitrem II and epoxyjanthitrem III were not completely resolved which necessitated further purification of epoxyjanthitrem II using an eluent of 19:1 acetonitrile-water, 10 mL/min. The preparative HPLC purifications detailed above resulted in five fractions each containing an individual epoxyjanthitrem compound. Final purification was achieved for each individual fraction by performing two preparative HPLC steps using 100% methanol, 10 mL/min as the eluent. NMR analysis showed each epoxyjanthitrem compound to be of high purity allowing a full NMR assignment to be made.

### 4.6. Mass Spectrometry

Semi-pure and pure compounds were analysed using an ESI-TOF mass spectrometer (MicroTOF; Bruker Daltonics, Billerica, MA, USA) operated with MicroTOF Control (Bruker Daltonics). Samples were resuspended in HPLC-grade MeOH (≤0.1 mg/mL) and introduced into the mass spectrometer via a syringe pump (3 µL/min). The positive ions were assessed using a capillary voltage of 4.5 kV and nebuliser pressure of 0.5 bar. Desolvation was carried out with a nitrogen flow of 4 L/min at 180 °C. The machine was calibrated with a solution of sodium formate (2 mM).

### 4.7. NMR Spectroscopy

Nuclear magnetic resonance spectra were recorded on a Bruker AVIII-400 NMR spectrometer. The chemical shifts were determined at 300 K and referenced to residual acetone in (CD_3_)_2_CO-d6 (^1^H 2.05 ppm, ^13^C 29.8 ppm). Samples were dissolved in (CD_3_)_2_CO-d6 (600 µL) before the 1D and 2D NMR spectra were acquired. Standard pulse sequences were used for HSQC, HMBC, COSY and NOESY experiments. HMBC spectra were acquired using parameter sets optimised for ^2-3^J ^1^_H-_^13^_C_ correlations.

Epoxyjanthitrem I (**1**): (4.10 mg, containing 2% epoxyjanthitriol) amorphous white powder, ^1^H and ^13^C NMR data; see Table 1 and Table 2, Figures S1–S6; HRESIMS *m/z* found 668.3536 (Figure S7) [M + Na]^+^ (calcd for C_39_H_51_NO_7_Na, 668.3558).

Epoxyjanthitrem II (**2**): (2.08 mg, containing 14.2% epoxyjanthitrem IV) amorphous white powder; ^1^H and ^13^C NMR data; see Table 1 and Table 2; HRESIMS *m/z* found 692.3927 [M + Na]^+^ (calcd for C_42_H_55_NO_6_Na, 692.3927).

Epoxyjanthitrem III (**3**): (3.75 mg) amorphous white powder; ^1^H and ^13^C NMR data; see Table 1 and Table 2; HRESIMS *m/z* found 694.4063 [M + Na]^+^ (calcd for C_42_H_57_NO_6_Na, 694.4078).

Epoxyjanthitrem IV (**4**): (2.26 mg) amorphous white powder; ^1^H and ^13^C NMR data; see Table 1 and Table 2; HRESIMS *m/z* found 736.4201 [M + Na]^+^ (calcd for C_44_H_59_NO_7_Na, 736.4184).

Epoxyjanthitriol (**5**): (2.76 mg) amorphous white powder; ^1^H and ^13^C NMR data; see Table 1 and Table 2; HRESIMS *m/z* found 604.3633 [M + H]^+^ (calcd for C_37_H_50_NO_6_, 604.3638).

## Figures and Tables

**Table 1:** ^1^H NMR Data ((CD_3_)_2_CO, 400 MHz) for epoxyjanthitrems I-IV (**1**–**4**) and epoxyjanthitriol (**5**) (δ in ppm, *J* in Hz).

Atom	1	2	3	4	5
1	9.86, s ^a^	9.87 s	9.83, s	9.86, s	9.84, s
5	2.68, m	2.64, m	2.65, m	2.67, m	2.65, m
	1.66, m	1.63, m	1.64, m	1.66, m	1.64, m
6	2.28, m	2.18, m	2.21 m,	2.26, m	2.25, m
	1.83, m	1.77, m	1.78, m	1.79, m	1.80, m
7	4.20, t (8.9)	4.32, t (9.0)	4.18, t (8.9)	4.16, t (9.0)	4.19, t (9.0)
9	3.48, d (9.4)	3.50, d (9.3)	3.39, d (9.1)	3.59, d (9.3)	3.35, d (9.0)
10	5.17, d (9.4)	4.04, d (9.3)	3.95, d (9.1)	5.26, d (9.3)	3.99, d (9.0)
11	3.55, s	3.56, s	3.50, s	3.51, s	3.52, s
14	1.63, m	1.65, m	1.61, m	1.63, m	1.63, m
	1.54, m	1.53, m	1.55, m	1.56, m	1.54, m
15	1.95, m	1.95, m	1.96 m,	1.98, m	1.96, m
	1.57, m	1.56, m	1.58, m	1.56, m	1.57, m
16	2.81, m	2.77, m	2.81, m	2.81, m	2.81, m
17	2.65, m	2.64, m	2.65, m	2.63, m	2.65, m
	2.35, dd (10.9, 13.0)	2.35, dd, (11.09, 12.8)	2.35, dd (10.9, 12.9)	2.35, dd (11.0, 13.0)	2.35, dd (11.1, 12.9)
20	7.18, s	7.18, s	7.18, s	7.17, s	7.18, s
23	7.39 s	7.38, s	7.38, s	7.38, s	7.39, s
25	1.32, s	1.32, s	1.32, s	1.32, s	1.32, s
26	1.16, s	1.14, s	1.13, s	1.17, s	1.14, s
28	1.13, s	1.18, s	1.28, s	1.20, s	1.19, s
29	1.14, s	1.28, s	1.25, s	1.18, s	1.22, s
30	3.08, dd (9.4, 15.6)	3.09, dd (9.3, 15.8)	3.09, dd (9.4, 15.6)	3.08, dd (9.2, 15.5)	3.09, dd (9.3, 15.6)
	2.66, m	2.64, m	2.65, m	2.64, m	2.65, m
31	2.83, m	2.83, m	2.86, m	2.86, m	2.87, m
35	5.93, d (2.9)	5.95, d (3.0)	5.94, d (3.0)	5.93, d (3.0)	5.94, d (3.0)
37	1.27, s	1.27, s	1.27, s	1.27, s	1.27, s
38	1.05, s	1.05, s	1.05, s	1.05, s	1.05, s
39	1.25, s	1.24, s	1.25, s	1.25, s	1.25, s
40	1.30, s	1.30, s	1.30, s	1.30, s	1.30, s
42	2.05, s				
43		5.58, d (6.2)	4.03, dd (2.6, 6.7)	3.92, d (6.6)	
44		5.17, d (6.2)	5.26, t (6.7)	5.20, t (6.6)	
46		1.70, s	1.71, s	1.68, s	
47		1.69, s	1.66, s	1.63, s	
49				1.99, s	
10-OH			4.29, s		
13-OH			3.29, s	3.42, s	3.31, s
27-OH					

^a^ s = singlet, d = doublet, t = triplet, dd = doublet of doublets, m = multiplet.

**Table 2:** ^13^C NMR Data ((CD_3_)_2_CO, 400 MHz) for epoxyjanthitrems I–IV (**1**–**4**), and epoxyjanthitriol (**5**).

Atom	1	2	3	4	5
2	154.9	154.9	155.0	154.9	155.0
3	51.7	51.7	51.7	51.6	51.7
4	43.2	43.3	43.2	43.2	43.3
5	26.9	27.1	27.0	26.9	27.2
6	28.7	29.5 ^a^	29.1 ^a^	28.8	29.3 ^a^
7	72.3	72.2	72.2	72.3	72.1
9	76.6	72.2	75.8	75.1	77.1
10	68.7	71.8	68.0	67.9	68.3
11	62.3	60.9	64.3	62.7	64.5
12	70.4	68.1	69.0	70.3	69.4
13	78.0	78.4	78.1	77.9	78.1
14	30.3	30.1 ^a^	30.4 ^a^	30.3 ^a^	30.3 ^a^
15	21.5	21.5	21.5	21.5	21.5
16	50.9	50.9	50.9	50.9	50.9
17	27.8	27.7	27.8	27.7	27.8
18	116.8	116.7	116.8	116.8	116.8
19	127.7	127.4	127.7	127.7	127.7
20	114.3	114.3	114.3	114.3	114.3
21	140.9	140.8	140.9	141.3	140.9
22	133.6	133.5	133.5	133.5	133.5
23	104.0	104.0	104.0	104.0	104.0
24	141.3	141.3	141.3	141.3	141.3
25	16.5	16.4	16.5	16.4	16.5
26	18.8	18.8	18.7	18.8	18.7
27	71.4	75.0	79.5	76.8	73.2
28	26.7 ^b^	28.8	23.9	24.5	24.3
29	26.6 ^b^	17.1	19.7	21.4	28.5
30	33.5	33.5	33.5	33.5	33.5
31	49.8	49.8	49.8	49.8	49.8
32	74.7	74.7	74.7	74.7	74.7
34	72.9	72.9	72.9	72.9	72.9
35	119.6	119.7	119.7	119.6	119.7
36	136.9	136.8	136.9	136.9	136.9
37	30.6	30.5	30.4 ^a,b^	30.4 ^a,b^	30.3 ^a,b^
38	22.4	22.4	22.4	22.4	22.4
39	32.4	32.3	32.3	32.3	32.4
40	30.6	30.5	30.4 ^a,b^	30.4 ^a,b^	30.3 ^a,b^
41	170.5				
42	21.2				
43		93.3	58.8	59.3	
44		124.0	122.2	124.0	
45		138.1	137.0	134.4	
46		25.5	25.8	25.7	
47		18.8	18.0	18.0	
48				170.2	
49				21.2	

^a^ Solvent obscured, identified in HSQC spectra. ^b^ Interchangeable.

## Supplementary Materials

The following are available online at https://www.mdpi.com/2072-6651/12/8/526/s1. Figure S1: ^1^H NMR spectrum of epoxyjanthitrem I in acetone-d6. Figure S2: Expansion of the 1.0–2.3 ppm region of the ^1^H NMR spectrum of epoxyjanthitrem I in acetone-d6. Figure S3: Expansion of the 3.6–10 ppm region of the ^1^H NMR spectrum of epoxyjanthitrem I in acetone-d6. Figure S4: ^13^C NMR spectrum of epoxyjanthitrem I in acetone-d6. Figure S5: Expansion of the 10–80 ppm region of the ^13^C NMR spectrum of epoxyjanthitrem I in acetone-d6. Figure S6: Expansion of the 100–180 ppm region of the ^13^C NMR spectrum of epoxyjanthitrem I in acetone-d6. Figure S7: HRESIMS of epoxyjanthitrem I (positive ion mode) containing a peak at m/z 668.3536 consistent with a molecular formula of C_39_H_51_NO_7_.
