# Supplementary material for: Identification and Structure Elucidation of Epoxyjanthitrems from Lolium perenne Infected with the Endophytic Fungus Epichloë festucae var. lolii and Determination of the Tremorgenic and Anti-Insect Activity of Epoxyjanthitrem I
Source: Toxins (Basel). 2020 Aug 17;12(8):526. doi: 10.3390/toxins12080526 (PMC7472112; doi:10.3390/toxins12080526)
Supplement: Supplementary file 1 [file toxins-12-00526-s001.pdf]

Supplementary Materials: Identification and Structure Elucidation of Epoxyjanthitrems from *Lolium perenne* Infected with the Endophytic Fungus *Epichloë festucae* var. *lolii* and Determination of the Tremorgenic and Anti-insect Activity of Epoxyjanthitrem I

Sarah C. Finch, Michèle R. Prinsep, Alison J. Popay, Alistair L. Wilkins, Nicola G. Webb, Sweta Bhattarai, Joanne G. Jensen, Allan D. Hawkes, Jacob V. Babu, Brian A. Tapper and Geoffrey A. Lane

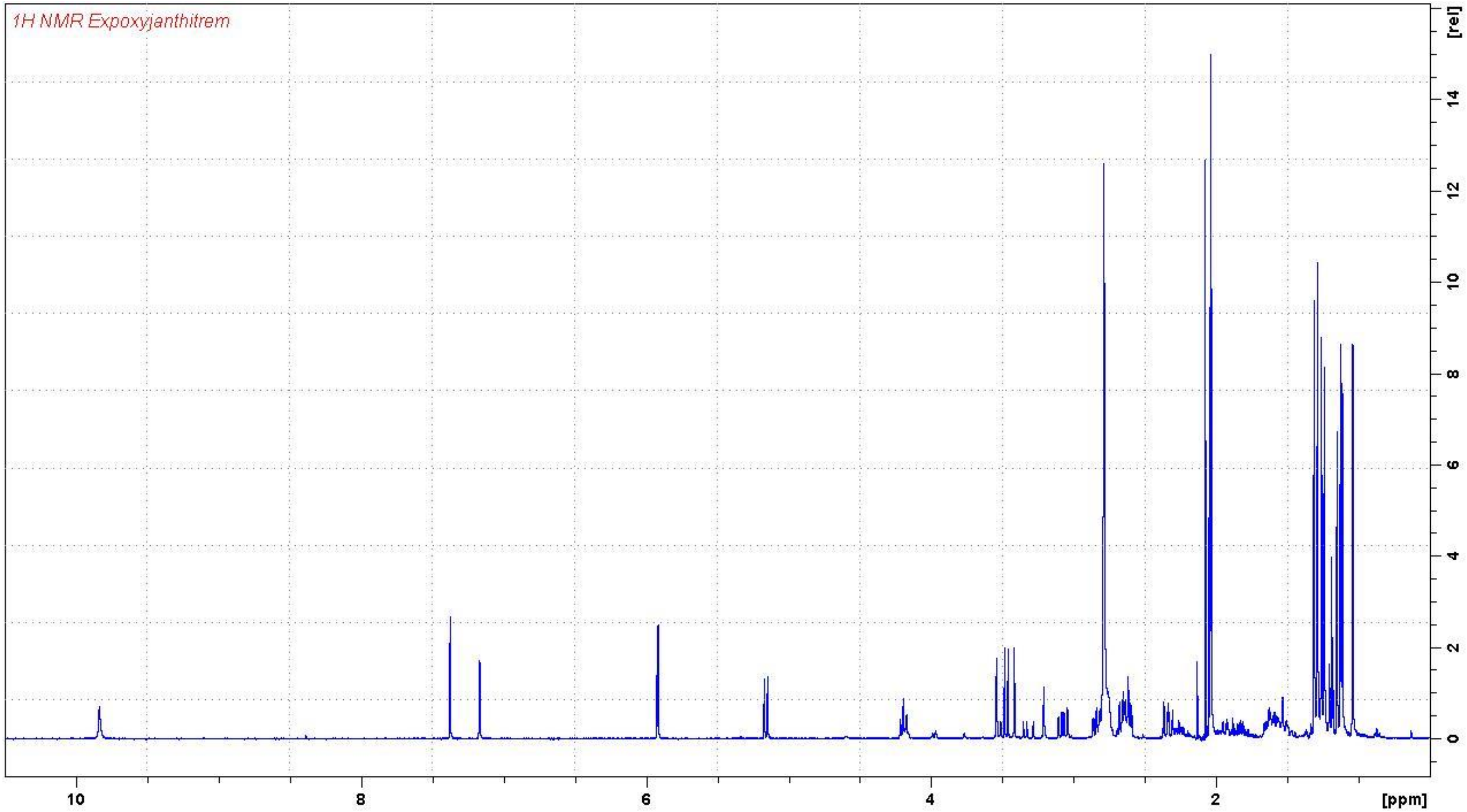

Figure S1. ^{1}H NMR spectrum of epoxyjanthitrem I in acetone-d6.

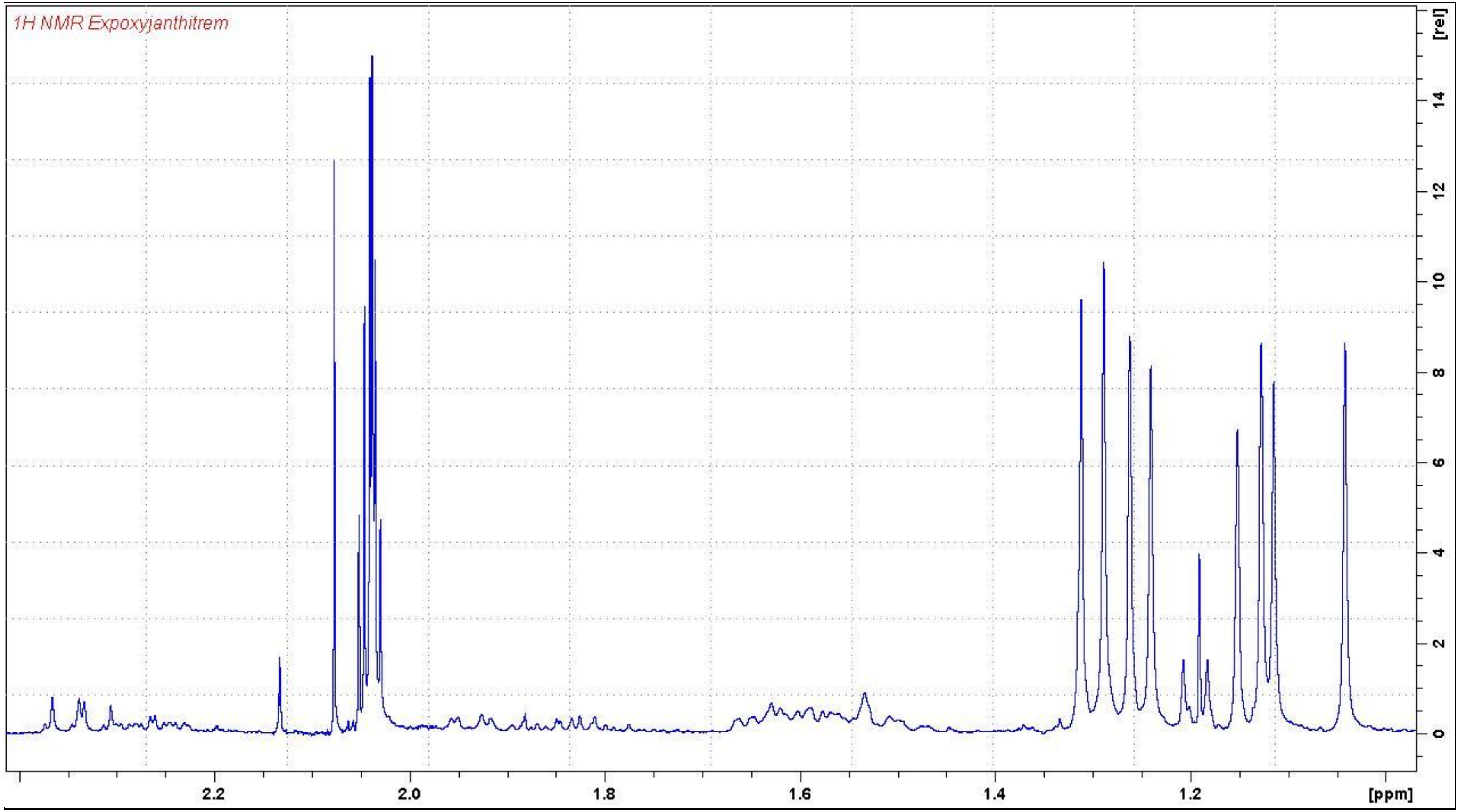

Figure S2. Expansion of the 1.0–2.3 ppm region of the ^1H NMR spectrum of epoxyjanthitrem I in acetone-d6.

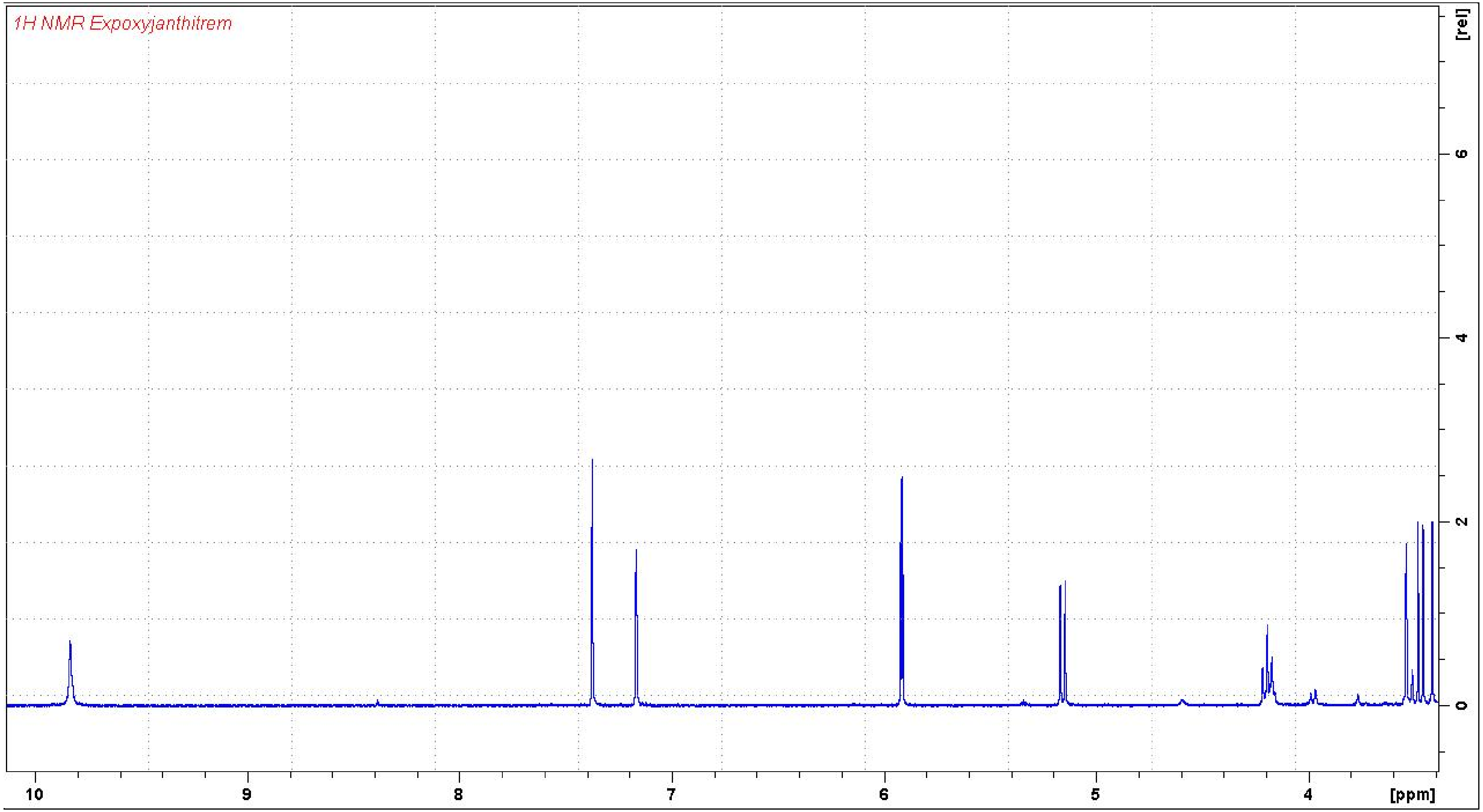

Figure S3. Expansion of the 3.6─10 ppm region of the ¹H NMR spectrum of epoxyjanthitrem I in acetone-d6.

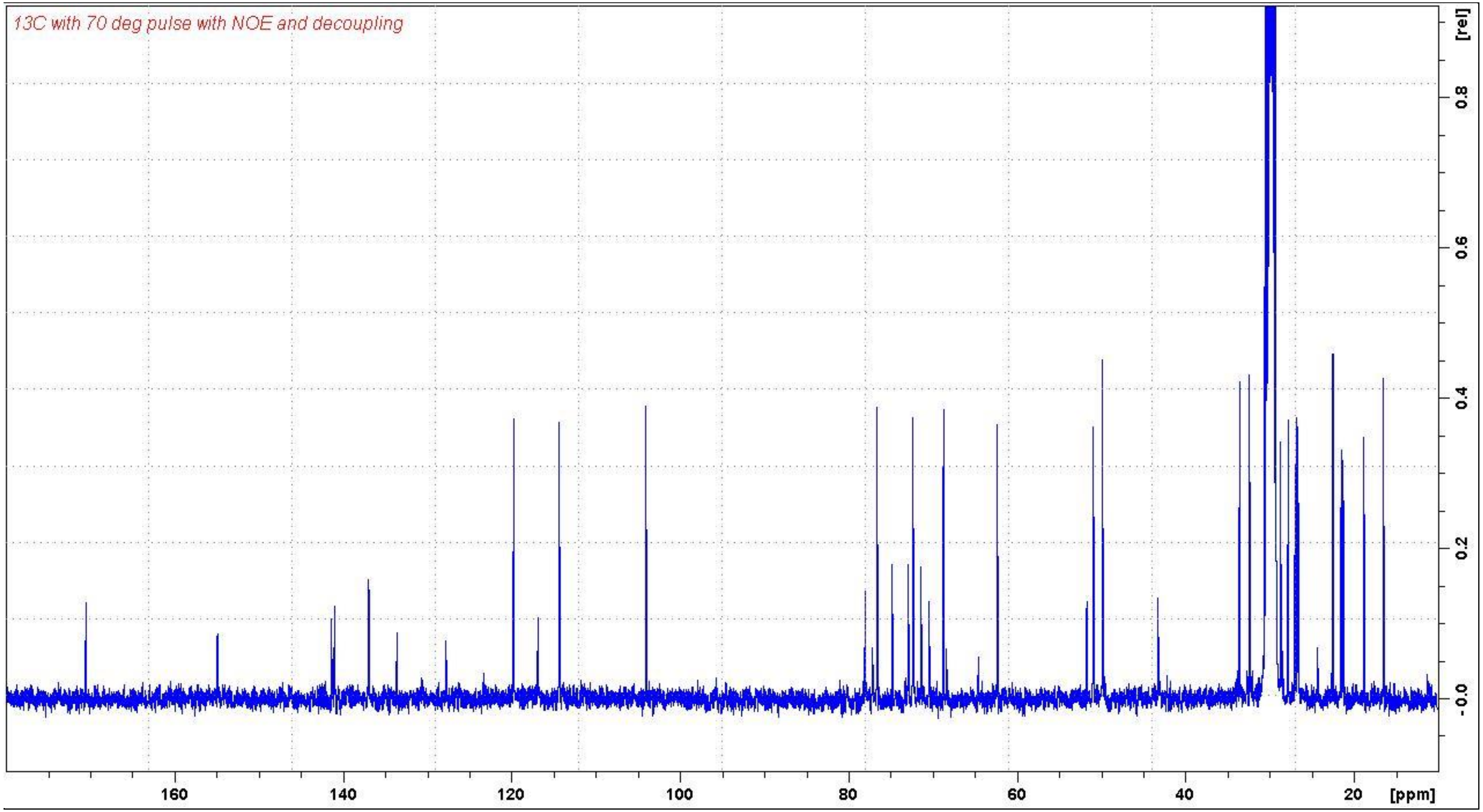

Figure S4. ^{13}C NMR spectrum of epoxyjanthitrem I in acetone-d6.

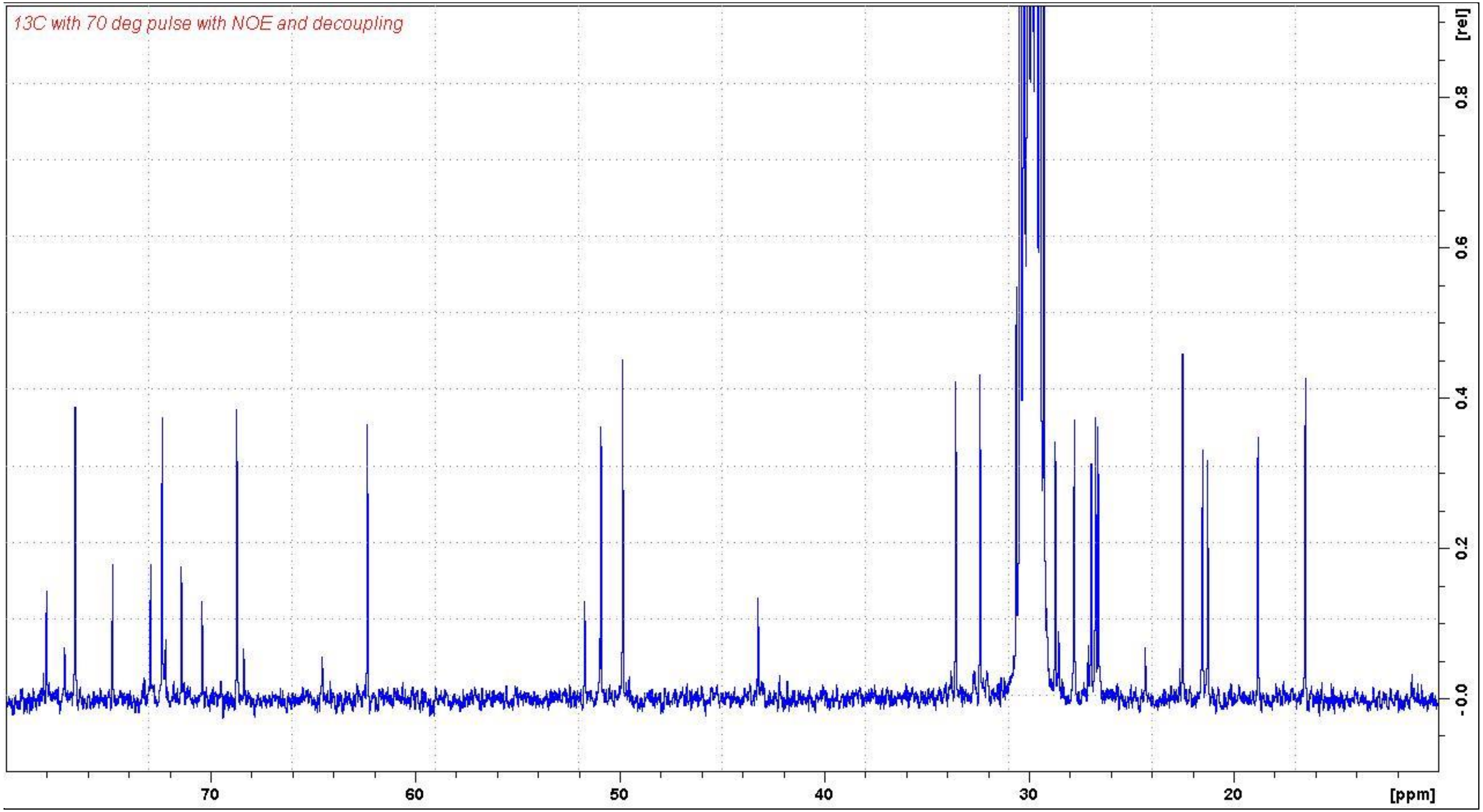

Figure S5. Expansion of the 10–80 ppm region of the ^{13}C NMR spectrum of epoxyjanthitrem I in acetone-d6.

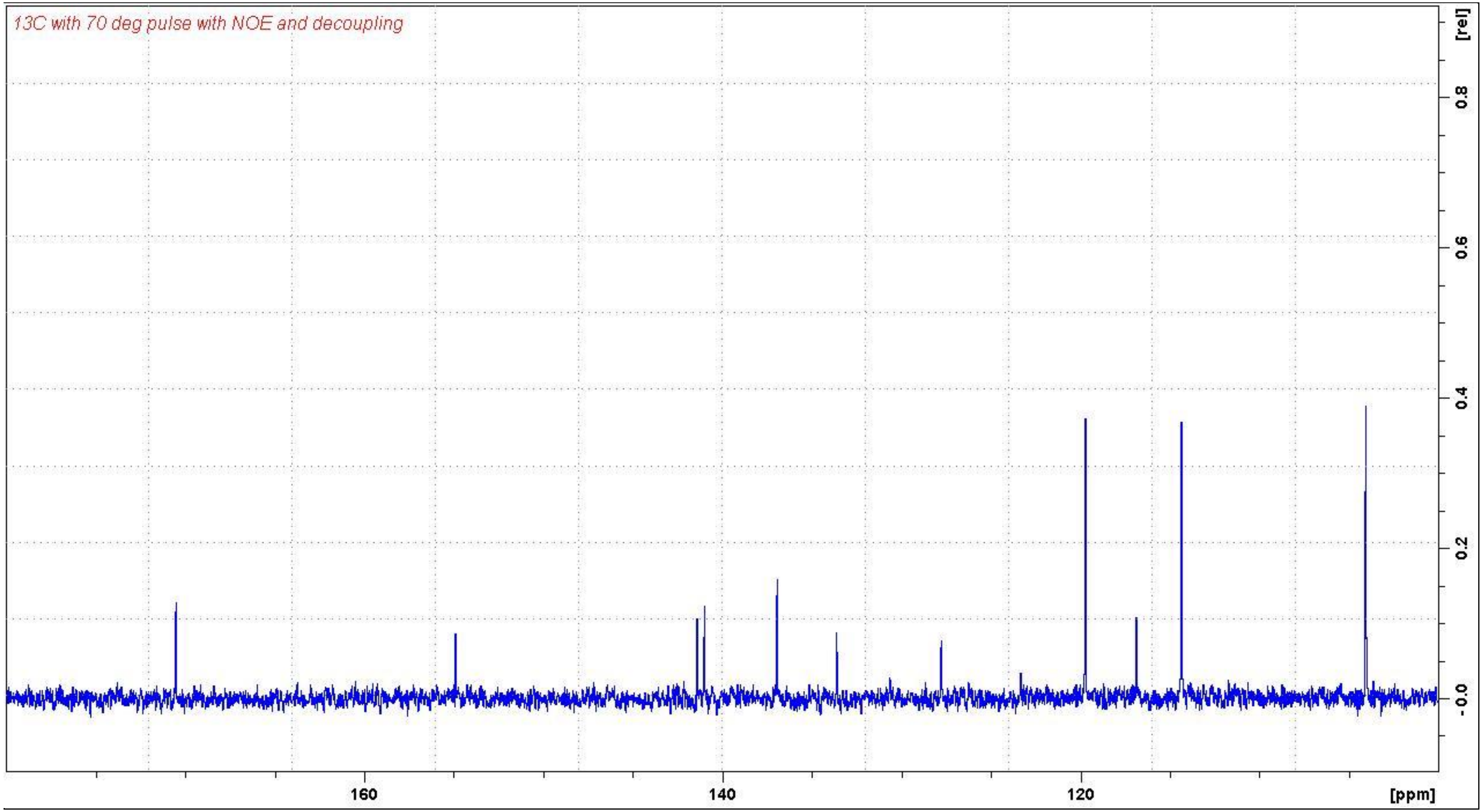

Figure S6. Expansion of the 100–180 ppm region of the ^{13}C NMR spectrum of epoxyjanthitrem I in acetone-d6.

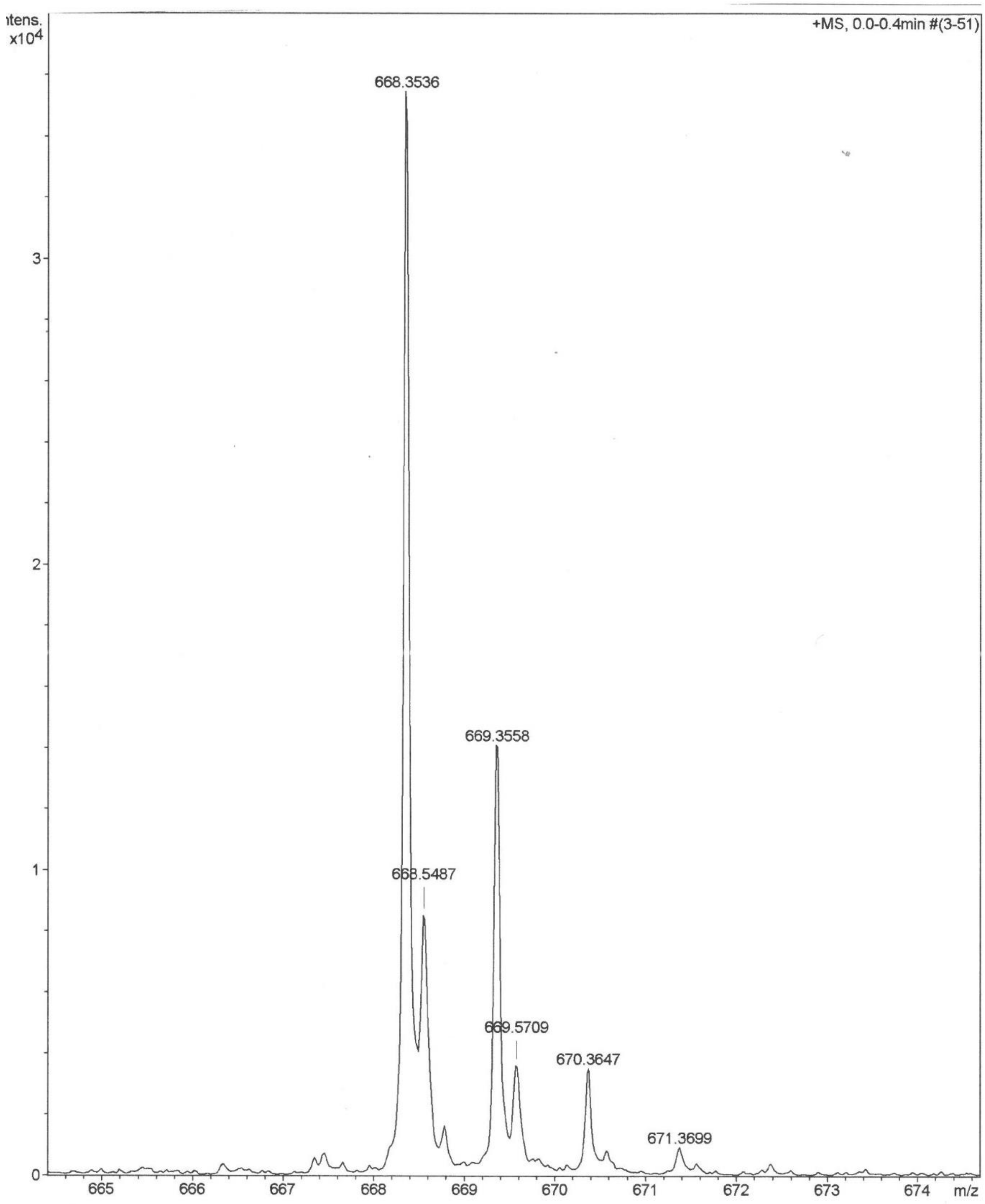

Figure S7. HRESIMS of epoxyjanthitrem I (positive ion mode) containing a peak at m/z 668.3536 consistent with a molecular formula of $C_{39}H_{51}NO_7$.
